# Post-Traumatic Stress Disorder (PTSD) Following Childbirth: Prevalence and Contributing Factors

**DOI:** 10.5812/ircmj.2312

**Published:** 2013-03-05

**Authors:** Zainab Shaban, Mahrokh Dolatian, Jamal Shams, Hamid Alavi-Majd, Zohreh Mahmoodi, Homeira Sajjadi

**Affiliations:** 1 Shahid Beheshti University of Medical Sciences, Tehran, IR Iran; 2 Department of Midwifery, Shahid Beheshti University of Medical Sciences, Tehran, IR Iran; 3 Department of Psychiatry, Behavioral Research Center. Shahid Beheshti University of Medical Sciences, Tehran, IR Iran; 4 Department of Biostatistics, School of Paramedical Sciences, Shahid Beheshti University of Medical Sciences, Tehran, IR Iran; 5 Faculty of Nursing and Midwifery, Department of Midwifery, Alborz University of Medical Sciences, Karaj, IR Iran; 6 Social Determinant of Health Research Center, University of Social Welfare and Rehabilitation Sciences, Tehran, IR Iran

**Keywords:** Natural Childbirth, Stress Disorders, Post-Traumatic Stress Disorder (PTSD), Incidence

## Abstract

**Background:**

Childbirth might be a traumatic event for some women.

**Objectives:**

This study was conducted with the objective of investigating the prevalence of Post-Traumatic Stress Disorder (PTSD) following childbirth.

**Patients and Methods:**

The study was designed using a descriptive correlation scheme. The participants were selected from the women referred to the healthcare centers affiliated with Zahedan University of Medical Sciences, Zahedan, Iran. Personal interviews were conducted with 600 women who were 6-8 weeks postpartum and had been undergone to this center for postpartum and child care.

**Results:**

One hundred and three (17. 2%) women had symptoms of PTSD following childbirth based on the PTSD Symptom Scale (PSS). The results of logistic regression analysis revealed a significant correlation between maternal occupation (P = 0.01), depression level (P < 0.001) and anxiety level (P < 0.001) with PTSD following childbirth.

**Conclusions:**

PTSD from childbirth occurs in some women. Early identification of risk factors should lead to early therapeutic intervention in the mothers at risk of PTSD.

## 1. Background

Anxiety disorders are one of the most common mental disorders. These disorders are twice as prevalent in women compared to men, which is attributed to the stressors (1). Post-traumatic stress disorder (PTSD) is an anxiety disorder that may follow an extremely traumatic stressor. The major symptoms of PTSD, according to the Diagnostic and Statistical Manual of Mental Disorders, 4th edition (DSM-IV) (2) include persistent replay of the traumatic event, persistent avoidance of stimuli associated with the event, numbing of general responsiveness, as well as symptoms of increased arousal. In various studies, childbirth has been recognized as a traumatic event and approximately 1.5% to 6% of women experience PTSD following childbirth (3-5). Factors like pregnancy complications, emergency caesarean, instrumental delivery, inadequate care during the labor (3, 6, 7), unwanted pregnancy, low socioeconomic status, history of infertility (8, 9), episiotomy, the severe pain experienced during the birth, postpartum complications (3, 5, 10), primiparous, preterm labor, parenting problems (3, 8, 11), cultural factors such as the importance of the baby’s gender, level of social support following childbirth (12-14), history of mental problems (3, 15) and stressor events of life (3, 16) make women susceptible to developing PTSD following childbirth. Consequences of this disorder may result in a vulnerability of the mother-infant attachment and have harmful effects on the infant’s cognitive development (9, 13, 17). The symptoms of this disorder are debilitating and affect the social, occupational, psychological, and communicative functioning of mother with her baby and family. Thus, awareness about the possibility of occurrence of this disorder following delivery, and recognition of its risk factors is an important issue for healthcare staff, especially obstetricians, who are in close contact with the mothers during the prenatal, intra- partum and postpartum periods.

## 2. Objectives

The author decided to investigate the prevalence of PTSD following childbirth among the women attending the healthcare centers affiliated with Zahedan University of Medical Sciences, Zahedan, Iran during the years 2009-2010.

## 3. Patients and Methods

Sampling of the current study was conducted from December 2009 to May 2010. The participants included 600 women who were attending the mother and baby units of the healthcare centers in Zahedan city, Iran to receive postpartum and child care services. The mothers who enrolled in this study were 6-8 week postpartum and had delivered a live child. The healthcare centers in this study were selected randomly using a multi-step method and based on the attendance days in these centers. The participants were selected on the basis of their availability, and after obtaining a written consent from each participant, the corresponding questionnaires were filled in the questionnaires included the customized screening form, designed by the researcher, containing demographic characteristics, obstetric factors (maternal and neonatal) and also mental and neurological factors. Validity and reliability of this form were assessed using the content validity and the test-retest methods, respectively. The coefficient of correlation obtained from 10 completed screening forms with one week interval which was 98%. Furthermore, in order to assess the mental health of the mothers, the depression and anxiety dimensions of the Symptoms Check List-90 (SCL-90) were used. The depression and the anxiety dimensions consisted of 13 and 9 items, respectively, and the answers were rated from zero to four on the basis of the Likert scale (Never, Rarely, Sometimes, Often, Always). In order to identify the level of depression and anxiety, the total scores of each test were divided by the number of the items related to the corresponding disorder. The resulting numerical values were attributed to different levels of the disorder as follows: values less than 1 as without disorder, values from 1 to less than 2 as a mild disorder, values from 2 to less than 3 as a moderate disorder, and values equal to or higher than 3 as a severe disorder. Validity of the depression and anxiety dimensions was obtained as 0.90 and 0.97 respectively and their reliability based on the test-retest method was determined as 0.85 and 0.75 respectively (11). Social support following childbirth was evaluated using the second section of Social Support Questionnaire of Winfield and Tiggemann, consisting of 6 items with “Yes” and “No” answers. The reliability and internal consistency of this questionnaire has been calculated 0.80 and 0.95 respectively (18). Post-traumatic Stress Disorder Symptom Scale (PSDS) was used for the purpose of evaluating the status of post traumatic disorder among the participants. This scale contained 17 items that diagnose PTSD according to the Diagnostic DSM-IV criteria and could be used to evaluate the after-effects of childbirth with a high degree of specificity. These items also assessed the severity of PTSD symptoms. The questionnaire contained three groups of items including: Re-experiencing (5 items); Avoidance (7 items), and hyper Arousal (5 items) which are scored on a 4-point scale from ‘‘not at all’’ to ‘‘5 or more times per week/very much’’. For a diagnosis of PTSD, a score of 1 or more was needed: for one of re-experiencing inquiries (questions 1-5), 3 of avoidance inquiries (questions 6-12), and for 2 arousal inquiries (questions 13-17) (12, 19). Validity and reliability of this scale, using concurrent validity and test-retest methods were determined as 0.91 and 0.74 (19) respectively. Likewise, its correlation level, using a diagnostic clinical psychiatric interview, was obtained as 0.86 (20). The data were analyzed using the SPSS version 17. In order to evaluate the parametric variables, independent sample t-test, and for non–parametric variables chi-square, Fisher's exact test and Mann-Whitney’s test were used, respectively. Logistic regression was used to assess the reciprocal effect between the factors associated with PTSD. The significance level of P value less than 0.05 was applied for all of the statistical tests.

## 4. Results

Of the 600 women under study, 358 women (59.7%) had re-experiencing symptoms, 141 women (23.5%), avoidance symptoms, and 299 others (49.8%), hyper arousal symptoms. Correspondingly, 103 women (17.2%) had symptoms of all of the mentioned three subscales and therefore have been diagnosed with PTSD. The results obtained from the analysis of demographic factors revealed that the mean age of women in the PTSD-diagnosed group was 27.21 years (SD = 6.2) and in the group without PTSD it was 26.7 years (SD = 6.41). Most of the participants had high school level education (29%) and were housekeepers (86.3%). The mean age of spouses in the PTSD group was 31.53 years (6.91) and in the non-PTSD the mean age was 31.60 years (SD = 6.91) while most of them were self-employed (64.8%) and had high school level education (32.5). The rate of PTSD following childbirth affected the working mothers more than homemaker mothers (OR = 2.86, P = 0.01, CI: 95%: 1.27-6.4). Other demographic variables did not correlate with risk of PTSD following childbirth. Analysis of the maternal variables did not correlate with risk of PTSD following childbirth between the two groups regarding parity, history of vaginal and cesarean deliveries, history of abortion, stillbirth, infertility and assisted reproductive treatments, mode of delivery, episiotomy, the use of analgesia/anesthesia, number of antenatal cares received, history of hospital admission due to pregnancy and postpartum complications, physical diseases and mother’s coping and expectations of childbirth. Mothers with an unwanted pregnancy (P = 0.003) and her spouse (P = 0.02), pregnancy complications (P = 0.03), postpartum complications (P = 0.001) and unpleasant experience of labor pains (P = 0.001) revealed increased PTSD following childbirth (Table 1).

**Table 1.** Correlations Between the Maternal Variables and PTSD

Maternal variables	No. (%)	P value
**Parity**		NS
Primiparous	199 (32.2)	
Multiparous	401 (66.8)	
**History of infertility**	35 (5.8)	NS
**History of stillbirth**	39 (6.5)	
**Mode of delivery**		NS
Vaginal	434 (72.3)	
Elective cesarean	52 (8.7)	
Emergency cesarean	114 (19.0)	
**Maternal experience of labor**		0.001
Good	103 (17.2)	
Moderate	170 (28.3)	
Bad	327 (54.5)	
**Unwanted pregnancy from Mother's point of view**	146 (24.3)	0.003
**Unwanted pregnancy from spouse's point of view**	121 (20.2)	0.02
**Antenatal complications**	329 (54.8)	0.03
**Postpartum complications**	239 (39.8)	< 0.001

Analysis of the neonatal variables in the two groups regarding gender, favorite of infant’s gender for parents, gestational age at delivery, birth weight of the infant, history of illness and hospitalization of the infant and history of birth defects did not correlate with risk of PTSD following childbirth. Nevertheless the variables of infant feeding with formula feeding (P = 0.03), existence of abnormalities in newborns (P = 0.03) and infant caring problems (P = 0.009) revealed an increase the rate of PTSD following childbirth (Table 2).

**Table 2.** Correlations Between the Neonatal Variables and PTSD

Neonatal Variables	No. (%)	P value
**Birth weight**		NS
Less than 2500 grams	48 (8.0)	
2500 grams and more	552 (92.0)	
**Baby’s gender**		NS
Girl	331 (44.8)	
Boy	269 (55.2)	
**Gestational age**		NS
Term	496 (82.6)	
Preterm	79 (13.2)	
Post-term	25 (4.2)	
**Infant feeding type**		0.03
Breastfeeding	500 (83.3)	
Formula feeding	33 (5.5)	
Both	67 (11.2)	
**Diseases of infant**	95 (15.8)	NS
**Abnormalities of infant**	5 (8.0)	0.03
**Mother’s satisfaction with Baby’s gender**	535 (89.2)	NS
**Existence of infant caring problems**	139 (32.2)	0.009

**Figure 1.**
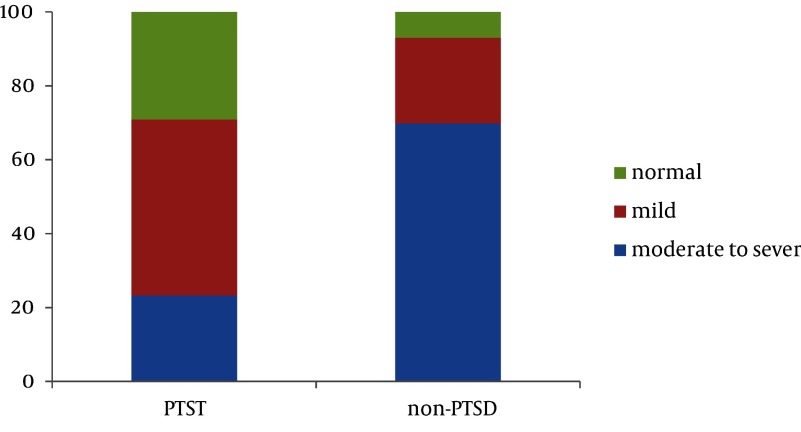
Rate of Depression Levels in PTSD and Non-PTSD Diagnosed Groups

Analyses of the neurological and psychological variables include the history of neuropsychiatric illness among the close family members and the presence of stressful life events over the last year did not correlate with the risk of PTSD following childbirth. Mothers with the history of neuropsychiatric illness (P < 0.001) and/or in her spouse (P < 0.01), the history of neuropsychiatric drugs use (P = 0.02) and/or in her spouse (P = 0.04), fear for injury or death of herself or her baby during the pregnancy (P < 0.001) as well as mothers with more depression and anxiety (P < 0.001) revealed an increase in rate of PTSD following childbirth. Social support level did not correlate with risk of PTSD following childbirth (P = 0.037). The results obtained from performing logistic regression and analysis of the corresponding variables’ reciprocal effects revealed that working mothers (P = 0.01), and mothers with more depression (P < 0.001) and anxiety (P = 0.01) had more of a tendency to suffer from PTSD following childbirth. The working women experienced PTSD 2.86 times more than the housekeeping mothers. The range of this increase with 95% confidence interval was 2.5 to 8.87. The results obtained from the analysis of depression and anxiety levels revealed that in comparison with non-depressed counterparts, the women with mild depression, experienced 4.7 times higher rates of PTSD following childbirth (CI: 95%, 2.5-8.87). This proportion for the women with moderate to severe depression levels was approximately 5 times (CI: 95% 1.91-13.3) (Figure 1).

Furthermore, the women with moderate to severe anxiety level, as compared to non-anxious women, suffered 3.35 times higher rates of PTSD following childbirth. The range of this increase with 95% confidence interval was 1.3 to 8.85.

## 5. Discussion

In this study, the prevalence of PTSD following childbirth in the female population of Zahedan was 17.2%. The reported universal prevalence of this disorder ranges from 1.5% to 6%. Andersen et al. (2012) (3) and Stramrood et al. (2011) (5) reported this value as 1-2%. Cigoli et al. (2006) (21) reported it as 1.25%, and Creedy et al. (2000) and Adewuya et al. (2006) reported the finding as 6.5% and 5.9%, respectively (6, 12). Social and cultural differences among various countries, different periods/procedures of identifying the women with PTSD in the postpartum period and differences in the instruments used in the evaluation can influence different results. The results related to demographic variables in the present study indicated that maternal age, the level of education, the spouses’ age, educational level and job, and the family’s economic situation did not increase the incidence of PTSD following childbirth. In the study of Adewuya et al. (2006), most women were 25-30 years old and their education was at high school level (6). Among these women, the demographic factors did not influence the risk of PTSD following childbirth, while Cigoli et al. (2006) suggested the social and demographic factors as influential features in the occurrence of this disorder (21). Occurrence of different results could be due to cultural and educational differences among different societies. These differences are crucial in the development of social skills and also in how people deal with life issues and manage stressful situations. Among the various demographic characteristics, mother's occupation presented a significant correlation with PTSD. The results of logistic regression analysis exhibited that the prevalence of PTSD following childbirth in the working women was 2.86 times more than that in the housekeepers. This situation could be related to the job stress at work. Mitani et al. (2006) suggested the existence of stress at workplace or the stressor nature of a job (22), and Van Der Ploeg and Kleber (2003) reported the level of support in family environment from the family members and in workplace from the colleagues, as crucial factors for increasing the vulnerability of people to mental disorders including PTSD (23). The results related to maternal variables revealed that an unwanted pregnancy for mother and spouse increased the prevalence rate of PTSD. Adewuya et al. (2006) expressed that unplanned pregnancy can play an important role in occurrence of PTSD (6) whereas in the study by Cohen et al. (2004) (10) this correlation was not validated. It seems that unplanned pregnancies and the reduced tolerance of maternal complications during the pregnancy as well as the reduction of support from the spouse because of his unhappiness with her pregnancy are associated with increased stress, anxiety and even depression levels during the pregnancy which, in turn, paves the way for development of such mental disorders as PTSD (6). Additionally, developing or the existence of pregnancy complications among most of the women in the present study and postpartum complications had increased the incidence of PTSD following childbirth. Various studies have revealedf that the occurrence of pregnancy complications could make women susceptible to developing PTSD through increasing problems and hardships for mothers, and also developing anxiety among them about the health of fetus and the outcome of pregnancy (24, 25). Brown and Lumley and (2000) reported that the occurrence of physical symptoms following childbirth makes the mothers mentally vulnerable and increases their risk of PTSD (26). Investigation of maternal experience of labor pains indicated that a severe experience of pain during childbirth had a strong correlation with the occurrence of PTSD. Soet et al. (2003) suggested the severe pain experience during labor and childbirth might be considered as a strong predictor of PTSD following childbirth (27). Bailham and Joseph (2003) also expressed that the severe pain experienced during the birth and maternal feeling of being obliged to endure this pain and labor can be an important factor in the occurrence of neurological problems and depression following childbirth (28). In addition, the social support of relatives especially the spouse, following childbirth has a significant impact on preventing their nervous problems and improving their mental status. The analyses of infant variables showed that the infant feeding method has an impact over the developing of PTSD. The concentration of gamma interferon and interleukin-10 in the blood of women who were using formula feeding for infant was lower when compared to the breast feeding women. This reduction would make them more susceptible to stress and traumatic events of life, resulting in the increased prevalence rate of depression and PTSD among them. Likewise, the blood level of prolactin hormone is an effective factor in reducing maternal stress (29). Women that use breast feeding report more positive mental condition, less anxiety and stress levels following childbirth and a positive perception about the childbirth events (30). In this study, the existence of infant abnormalities increases the risk of PTSD in mothers. Nagata et al. (2008) concluded that observation of abnormities and defects in newborns is a highly disturbing factor for mothers and causes mental disorders in some, while eliciting PSTD in others (31). In addition, the mothers’ involvement in treatment procedures and observing the recovery of their baby are effective factors in reducing the severity of these symptoms. Moreover, the results of investigating the neonatal factors showed that the existence of infant caring problems was associated with the development of PTSD. This factor was assessed by evaluating the mother’s perception about her infant’s status of sleeping, nutrition and crying. The mothers who reported their infant’s feeding and sleeping status less than their expectations and the infant’s crying duration as more than expected had problems with caring their babies (23.2%). It seems that women with the fear of not having successful breast feeding and inability to control their baby’s crying experienced more anxiety level during the postpartum period. There is also the stress of baby care, the resultant sleep deficiency and mother’s malnutrition which can worsen the mother’s mood condition (32, 33). The results related to neuropsychological variables indicated that the fear of injury or loss of self or infant causes mothers to experience PTSD. Czarnocka and Slade (2000), in the study of women with PTSD found that these mothers experience great fear about their health and their baby during pregnancy (34). Other studies have presented that the fear of injury or death of infant during the labor and childbirth is an important predictor for psychological problems following childbirth (35, 36). Söderquist et al. (2006) indicated that the history of neurological disease of mothers and their spouses as effective factor on PTSD during 1-11 months postpartum (15). Britton (2005) also expressed that history of personal or family depression, anxiety, and bipolar disorder may turn into severe mood postpartum (37). According to logistic regression, more depression and anxiety can increase the risk of PTSD in mothers and women with mild depression experience PTSD, 4.7 times more than normal, and women with moderate to severe depression experience PTSD nearly 5 times higher than the non-depressed women. Furthermore, women with moderate to severe anxiety are 3.35 times more vulnerable to PTSD than the non-anxious women. The likelihood of a person to be at risk of this disorder increases with the severity of depression and anxiety. N number of studies has been done on the biological causes of PTSD. Some of these studies are concentrated on the neuroendocrine dysfunction caused by a stressful event. Following a stressful event, the activity of neuroendocrine system increases and a drastic reduction occurs in the level of neurotransmitters such as norepinephrine and dopamine (1). Besides that, the activity of hypothalamus-pituitary-adrenal axis increases which is accompanied by the increase in cortisol hormone level. These endocrine changes cause anxiety and PTSD in the cases (38). According to Cowen (2002), depressed women experience reduction in the level of neurotransmitters and an increase in cortisol hormone level. The more intense is one’s depression, the more endocrine changes occur (39). Several studies have reported that following a trauma, depression increases as an important factor in developing PTSD (27, 40). The results of this study did not reveal an influence of social supports on the incidence of PTSD following childbirth. Cigoli et al. (2006) believed that there is a connection between low levels of social support from family members and medical personnel and experience of PTSD symptoms (21) while the findings of Calhoun et al. (2002) did not confirm any association of this agent with PTSD (41). Furthermore, the existence of cultural and social differences in interpretation and quality of support and paying more attention to the physical support instead of psychiatric and emotional support from a spouse and relatives of the mother following childbirth can be effective considerations in the occurrence and varying levels of these differences. Based on our findings, it can be concluded that pregnancy, childbirth and adaptability to the newborn are the most sensitive stages in women’s lives. One of the most important causes of the development of psychological problems in women is the experience of emotional stress and anxiety during pregnancy, childbirth and postpartum. Psychological disorders and the related complications have harmful effects on the quality of mother’s communication with infant and the infant's cognitive development. It also could disrupt a person’s social functions, occupation and individual relationships with other family members. Therefore, midwives should be aware of the possibility of these disorders and related factors in order to take preventive measures. Screening and on-time treatment of affected mothers is needed to prevent the occurrence of long-term complications.
